# Novel approaches to communication skills development: The untapped potential of qualitative research immersion

**DOI:** 10.1016/j.pecinn.2022.100079

**Published:** 2022-08-26

**Authors:** Amy S. Porter, Cameka Woods, Erica C. Kaye, on behalf of the QUEST Working Group, Taylor Aglio, Jacob Applegarth, Kelly Bien, Tharwa Bilbeisi, Emma Chow, Katie Greer, Rachel Huber, Ashley Kiefer Autrey, Sarah Rockwell, Marta Salek, Melanie Stall, Mariela Trejo, Yenny Yang, Kristina Zalud

**Affiliations:** a St. Jude Children’s Research Hospital, Memphis, TN, USA; b Oakland University William Beaumont School of Medicine, Royal Oak, MI, USA (Jacob); c University of Memphis, Memphis, TN, USA; d Rhodes College, Memphis, TN, USA; e University of California Davis Children’s Hospital, Sacramento, CA, USA; f Children’s Hospital of New Orleans, New Orleans, LA, USA; g Emory University, Atlanta, GA, USA; h University of Texas Southwestern Medical Center, Dallas, TX, USA; i University of Maryland School of Medicine, Baltimore, MD, USA; j University of Tennessee Health Sciences Center, Memphis, TN, USA; k St. Louis Children’s Hospital, St. Louis, MO, USA; a St. Jude Children’s Research Hospital, Memphis, TN, USA

**Keywords:** Communication training, Medical education, Qualitative research, Communication research

## Abstract

**Objective:**

Participation in qualitative research, particularly analysis of recorded medical dialogue, offers real-time, longitudinal immersion that can strengthen clinical trainee communication skills. The study objective was to explore how qualitative research participation impacts clinical trainees’ self-perceived communication skills development and practice.

**Methods:**

In this study, a 17-member multidisciplinary working group of child life specialists, advanced practice providers, undergraduate/medical students, residents, fellows, attending physicians, social scientists, and career researchers with recent qualitative and communication research experience assembled to discuss this topic using a structured discussion guide. Content analysis was used to identify concepts and themes.

**Results:**

Three key themes characterizing the impact of qualitative research participation on aspiring clinicians’ communication skills development and practice arose – the 3Cs: (1) **C**onnection, therapeutic alliance, and accompaniment; (2) **C**larity and prognostic communication; (3) **C**ompassion, empathy, and understanding. Participants emphasized that qualitative research learning improved their understanding of patient/family lived experiences, preparing them for future clinical encounters, strengthening their emotional intelligence, and promoting self-care, resilience, and professional affirmation.

**Conclusions:**

Immersion in clinical communication through participation in qualitative research is an under-utilized resource for supporting clinical trainees in communication skills development.

**Innovation:**

The process of collaborative knowledge production through the collective exploration of an a priori question related to group members’ collective experiences is methodologically innovative. Further, re-thinking qualitative research participation as an underutilized educational opportunity is pedagogically novel, and leaders in medical education and qualitative research should collaborate to realize the potential of this teaching tool.

## Highlights

• Qualitative research participation offers immersion in clinical communication.
• Participation impact characterized by the 3 C’s: 1) Connection, 2) Clarity, 3) Compassion.
• This is an under-utilized medical education resource for communication skills development.
• Medical education and qualitative research leaders should collaborate.

## 1 Introduction

Communication training for clinical trainees often involves single timepoint simulation as a “gold standard” for practicing navigation of challenging conversations [[1], [2], [3], [4], [5], [6], [7], [8], [9], [10], [11], [12]]. Due to time, staffing, and resource constraints, medical educators face challenges realizing high volume of real-time communication learning opportunities [1]. Clinicians-in-training are exposed infrequently and inconsistently to in-depth, communication-heavy encounters between clinicians and patients and their families during difficult moments in the illness course [5,13,14]. As a result, trainees lack robust opportunities to witness communication and consider which modeled approaches they want to integrate into their own communication toolboxes. Further, depending on supervisory ratios, trainees may not have sufficient opportunities to observe clinicians with a range of emotional dexterity skills to learn and reflect on how (or how not) to communicate during challenging medical encounters.

Healthcare communication science researchers have amassed large repositories of recorded medical dialogue to answer questions about best practices for communication between patients, families, and healthcare professionals; however, little precedent exists for collaboration between communication researchers and medical education leaders to optimize use of this under-utilized resource to offer learners opportunities for developing communication skills through participation in communication research. Existing literature explores how guided reflection activities such as “The Healer’s Art” and other self-contemplative didactics positively impact trainees’ communication skills, empathy, self-awareness, and overall clinical practice [[15], [16], [17]], yet the potential educational value and impact of qualitative research experiences on trainees’ learning and communication skills remains understudied and poorly understood.

To address this knowledge gap, we convened a multidisciplinary working group of students, clinicians, and researchers to consider the question: *“How does engaging in qualitative communication research (i.e., listening to audio recordings and/or reading transcripts of recorded clinical encounters) impact trainees as professionals (both clinicians and researchers) and as individuals holistically?”* The Qualitative research as Education for Students and clinicians-in-Training (QUEST) working group comprised individuals affiliated with a communication research lab within an academic institution who each had recent experiences participating in qualitative research on topics related to communication. The working group examined whether engaging in qualitative research involving patients and families could influence the way students and clinicians-in-training learn and practice communication. In this article, we summarize findings from the QUEST working group and propose immersion in qualitative research datasets as an innovative alternative or complement to standardized simulated communication skills training.

## 2 Methods

In this study, we used an adaptation of autoethnography to bring together a team of authors with common experiences related to qualitative research participation, collectively share our perceptions and generate reflective data about our experiences with qualitative research, and collaborate with one another to analyze the data and present our insights. In traditional autoethnographic methods, an individual uses a reliable process to generate data from their own experience, observations, and reflections and then reflects on and synthesizes these data to inform a larger context [18]. Koopman et al describes autoethnography as the ultimate form of reflexivity, a mechanism by which to explore personal perceptions, values, and beliefs through the lens of lived experience, culture, and self-other interactions [19]. In this project, our authorship team wished to gain deeper insights into the potential influence of qualitative research participation on communication education for students and clinicians. In deciding to study ourselves, we developed a modified form of autoethnography, which we describe below. This paper reports the findings from the QUEST working group with all group members represented as authors; there were not separate groups representing “researchers” and “study participants,” but rather one collaborative group working together to explore an *a priori* question related to our collective experiences. As such, the project did not require IRB approval.

The authorship team convened as the QUEST working group, comprising a 17-member group of students, staff, and faculty with recent qualitative communication research experience, including undergraduate/medical students, residents, fellows, child life specialists, advanced practice providers, and clinical research staff. Within the Quality of Life and Palliative Care Research Division, all learners who had participated in communication research by listening to recorded medical dialogue or reading transcripts of interviews with patients, families, and clinicians at a particular academic institution over the past 3 years were invited via email to participate (n = 21). No exclusionary requirements were applied. Though all invited individuals expressed interest in joining the QUEST working group, a total of 17 people ultimately participated. Individuals agreed to participate in working group conversations by responding in writing to the email invitation. All working group members had participated in analysis of at least one qualitative data set related to communication, with most participating in qualitative research for at least one year (although outliers included 1 member with a 2-month qualitative research elective and 1 member with 5+ years of qualitative research participation). Most of the group was comprised of nursing/medical trainees (e.g., undergraduate, graduate, nursing, and medical students; fellows; n=13); the group also included 2 clinical research staff who engage in communication with patients and families, 1 child life specialist who participated in qualitative research, and 1 clinician-researcher who oversees qualitative research studies. Members’ training, roles, and experiences interfacing with different types of qualitative data are presented in Table 1.

**Table 1:** QUEST working group member characteristics.

QUEST member	Degree(s)	Role while participating in qualitative research	Qualitative research experience	Role in collaborative knowledge production
T.A.	MD	Fellow	Written transcripts & audio recordings	Virtual meeting
J.A.	MD	Medical student	Written transcripts	Email response
K.B.	RN, CRA	Clinical research associate	Audio recordings	Virtual meeting
T.B.	PhD	Graduate student	Audio recordings & written transcripts	Email response
E.C.	n/a	Undergraduate	Audio recordings	Virtual meeting
K.G.	CLS	Child life specialist	Written transcripts	Email response
R.H.	RN	Nursing student	Audio recordings	Virtual meeting
E.K.	MD, MPH	Physician, researcher	Written transcripts & audio recordings	Virtual meeting
A.K.	MD	Fellow	Written transcripts	Virtual meeting
A.P.	MD, PhD	Resident, Fellow	Written transcripts & audio recordings	Virtual meeting
S.R.	MPH, MSN, APRN, CPNP	Advanced practice provider student	Audio recordings	Virtual meeting
M.S.	MD	Fellow	Written transcripts	Virtual meeting
M.S.	MD	Fellow	Audio recordings	Virtual meeting
M.T.	BS	Medical student	Written transcripts	Email response
C.W.	BS, CRA	Clinical research associate	Written transcripts & audio recordings	Virtual meeting
Y.Y.	BS	Medical student	Written transcripts	Virtual meeting
K.Z.	MD	Medical student, Resident	Written transcripts	Email response

The three lead authors crafted a semi-structured working group discussion guide, with iterative revisions to refine questions for content and language. Supplemental Figure presents the guide, encompassing a semi-structured outline of questions prompts and probes to organize and support cooperative conversation. Working group members were encouraged via email to join a virtual 120-minute discussion; those who could not attend were given an opportunity to respond to the questions in writing. A physician-medical anthropologist with training and expertise in group engagement facilitated the virtual discussion. Twelve QUEST working group members, including the three lead authors, attended the recorded virtual session using WebEx (an online platform for virtual group meetings). The conversation introduction included reminders about the importance of reflexivity and how participants’ positionality influences (and may bias) perspectives. Throughout the virtual discussion session, each participant remained engaged and interacted with most question probes, yielding multiple responses for each question. A working group format was used intentionally to explore the targeted question, given the positive potential for group dynamics to help with idea generativity and allow reflections to build upon others’ thoughts and observations [20,21]. Most working group members were students and trainees or clinical research staff, interacting within similar hierarchical tiers. Recognizing the potential for hierarchy to constrain conversation, the one faculty member in a supervisory position observed quietly, engaging only when asked a direct question by another working group member.

Five working group members were unable to attend the virtual discussion due to their training schedules, and they wished to participate in the exploratory question. To ensure inclusion of their voices and perspectives, they were given an opportunity to provide written reflection responses to each item in the structured discussion guide; these lengthy responses were shared via email to contribute their perspectives to the conversation.

Following data generation, the three lead authors initially conducted memo-writing of the recorded discussion and written responses to begin reflecting on and discussing emerging patterns in working group conversation content [22]. Memo-writers purposefully represented different perspectives from a current clinical trainee, a research staff member, and a faculty member, with iterative discussions held in person and via email to explore how different viewpoints influenced reflections in memos and examine internal biases shaping thoughts and assessments. Content analysis was used to synthesize working group transcripts as this method provides a rigorous process for identification of concepts and themes within text. As concepts were inductively generated via memo-writing, findings were shared with all QUEST members for iterative reflection and input. The QUEST working group collaborated to synthesize and review key themes, with cycles of review and refinement among authors [23,24]. The final report was presented to the working group for member-checking [25], with confirmation from all authors that thematic findings reflected the comprehensive content of working group discussions.

## 3 Results

**Fig. 1:**
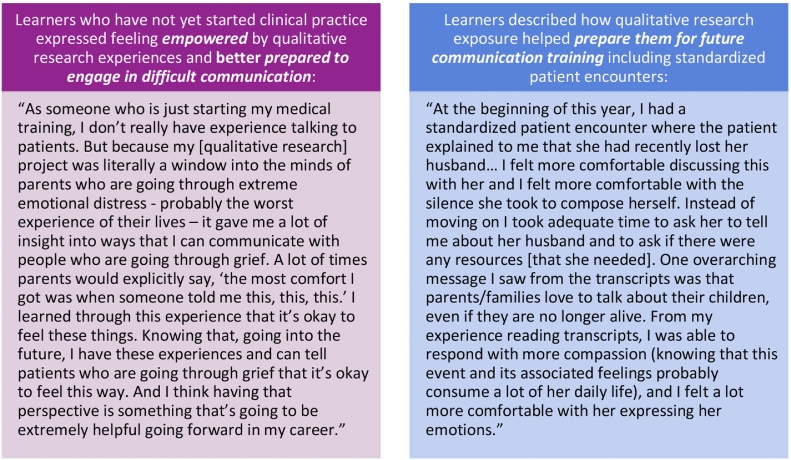
Influences of qualitative research immersion on learner communication skills.

Working group members consistently emphasized the value of immersion in qualitative research, highlighting the utility of engagement with audio recorded and/or transcribed clinical encounters that included challenging communication scenarios (Fig. 1). Nearly all members described the impact of qualitative research experiences on their personal communication skills and practice, and two driving themes emerged to characterize the “value added” by qualitative research: 1) the tangible benefits of exposure to difficult medical communication prior to real-life encounters; and 2) the potential for long-lasting impact and sustained influence of qualitative research experiences on future clinical practice, including three specific impacts on communication skills (“the 3Cs”).

### 3.1 Immersive learning prior to real-life training and practice

For many working group members, communication challenges in healthcare were largely hypothetical prior to their participation in qualitative research. Coding real clinical encounters as part of qualitative research revealed the complexity of interpersonal communication and offered lessons for how to navigate difficult conversations with actual patients and families: “I really saw models of what this actually looks like and how do patients and their families respond to different styles.” Authors with limited previous exposure to clinical encounters also shared how immersion in raw qualitative data helped them recognize the emotional intensity experienced by patients, families, and clinicians:

“Listening [to audio-recorded medical dialogue] really helped me to understand how much tension there can be in a room… Just listening to long pauses of silence helped me understand that [prognostic communication] can be really challenging emotionally, both on the clinician side and the family and patient side, how challenging it can be to navigate that both as a parent and as a clinician.”

Another member described how her participation in qualitative research as a medical student informed her future practice as a resident:

“I began intern year in the intensive care unit and had several patients die within my first two to three weeks of residency. Communicating with these families about the goals or priorities of their loved one and then having to tell them when that person had died required attention to detail, meticulous word choice, and rapport building. All of these skills were taught or honed by the coding experience.”

Universally, working group members highlighted how exposure to “real” clinical encounters offered them unique experiences to observe communication skills and reflect on interpersonal dynamics that they could carry forward into their future clinical practice.

### 3.2 Sustained influence on future clinical practice

Overall, working group members agreed that participating in qualitative research had a greater impact than they anticipated on the way that they provide clinical care. One child life specialist explained specifically how real clinical encounters still shape her everyday clinical practice:

“I was not anticipating the coding experience [would] play such an influential role in my day-to-day clinical practice. The process has made me more reflective in my everyday interactions with patients and families, as I have various narratives to refer back to, and [they] are typically at the forefront of my thoughts when interacting with families now.”

A palliative care physician explained how specific clinician-patient or clinician-family interactions persist in a clinician’s mind through years of clinical practice: “Some of the quotes stick with you and influence your practice.” Many working group members echoed this idea of staying power – conversations witnessed through reading transcripts or listening to audio recordings remained impressed on their minds as reference points for choosing language, reflecting on clinical encounters, and remembering the complexities of patients’ and families’ experiences.

The working group also identified three key themes characterizing how immersion in qualitative communication research influenced aspiring clinicians’ self-perceived communication skills development – the 3Cs (Table 2): 1) **C**onnection, therapeutic alliance, and accompaniment; 2) **C**larity and prognostic communication; and 3) **C**ompassion, empathy, and understanding.

**Table 2:** 3Cs: Key themes characterizing the impact of qualitative research participation on learners’ communication skills development.

Lesson learned	Example quotation
**C**onnection – establishing connection, manifesting accompaniment, & building therapeutic alliance	
Importance of careful listening	“It really opened my eyes to the importance of being a really good listener. I don’t always know how to respond or the right thing to say. But sometimes when I feel that way, just being silent and just listening instead of feeling like I have to have something to say.”
Value of silence	“I have embraced silence a lot more than I ever had…I find myself at different times actually counting in my head how long I have been silent even if it’s not a situation in which I need to be…[listening to recordings has] very much ingrained in me that silence is okay and can be comfortable.”
Importance of affirming emotions	“It was interesting to hear some of the same themes emerge for various parents [in the written transcripts] and made me think of how impactful it may be to validate the participants’ emotions/feelings by sharing those common themes with them.”
Importance of establishing rapport	“As a newer clinician, I was very pleasantly surprised by how much bond there was between the provider and the patient and the family across the recordings…Listening to that bond and what the family is going through in their private life…made me aspire to reach that.”

**C**larity – achieving clarity and effectively communicating prognosis	
Value of direct, clear prognostic communication	“On my first day [as a resident] in the Pediatric Intensive Care Unit (PICU), one of my patient’s family [members] was going through the absolutely heart-wrenching decision to withdraw care…Our attending thoughtfully gave some pointers regarding communication with her family, saying that sometimes people felt the need to say things like ‘there's always hope,’ which he thought would be detrimental to their decision process. I don't think that the original sentiment is wrong; hope may be reframed. I agreed that this statement wouldn't be language I would utilize, largely based on my prior qualitative work regarding prognostic communication in the setting of bad news. I felt this family deserved the news in a clear and kind way that their child would never be the same and this problem could not be fixed.”
How to carefully and intentionally frame “bad news” of all varieties	“I think in working with data from families who were receiving bad news, I have also come to conceptualize that bad news comes in a variety of forms. Sometimes it is a life-changing diagnosis. Other times it’s staying another day when you thought you could finally go home. I want to be emotionally present in both kinds of conversations.”
Heightened awareness of the effects of language on patients/families	“Coding forced me to consider the significance of communication [at] a very granular level. It showed me that even a few select words or a singular phrase has the ability to change how a response could be received. This idea was further reinforced during reconciliation of codes after coding in silos. Hearing how another individual, someone who has chosen the same career field and in several ways is fairly similar to me, interprets a phrase in a drastically different way was eye opening.”

**C**ompassion – caring compassionately through empathy and understanding	
Empathy, deeper understanding of patients’/families’ experiences, how to witness people’s lived experiences	“Getting the whole story… The grief journey, the time spent at the hospital, is honestly only one small part of it. A lot of these people have had to uproot their entire lives to come to [the hospital]. Their relationships with everyone around them change. They have to give up their job; they lose friendships; they gain new ones… Getting that comprehensive picture of everything that’s going on in their lives – it’s just really profound.”
Compassion for patients and families	“Reading transcripts and gaining insight on a tragic loss, such as the death of a child, from bereaved parents has made me more sensitive to patient concerns and communication. In many of the transcripts I read, I saw how one single interaction with a provider can have a lasting impact on a patient/family, and I hope to remember this as I navigate the clinical setting next year. Small acts of kindness can go a long way. I think that it has also made me a more compassionate individual to understand how people have their daily battles which may manifest in different ways… I might not understand first-hand what a person is going through behind the scenes and I might not know all the baggage they carry, but if I can offer some type of kindness to lighten their load, I want to try my best to do that. I hope that on days when I feel burned out or tired I can remember this and treat all my patients with the utmost respect and compassion they deserve, knowing that everyone has struggles they are dealing with.”

### 3.3 Skills for aspiring clinicians: connection

Working group members described how witnessing clinicians’ approaches for establishing connection and building therapeutic alliance with patients and families helped them learn how to develop their own skills. Many mentioned the importance of listening carefully to patients and families, as well as the value of silence:

“This experience helped me further develop active listening skills. I think silence is something that often makes people uncomfortable; however, this experience made me realize how many families…want and need a space to process and have others actively listen to their thoughts and emotions. It was very humbling to be a part of that process.”

As detailed in Table 2, others discussed how they came to realize that affirming patients’ and families’ emotions is essential to establishing therapeutic alliance and how witnessing clinicians establish rapport with families led them to aspire to do the same in their own clinical practice.

Several working group members contemplated the sensation of privilege upon entering what felt like experiencing prognostic communication *with* the patient and family – accompanying them through the illness trajectory. One nurse practitioner explained that, despite having been a bedside oncology nurse prior to participating in qualitative research, listening to recorded conversations was the first time she had been “in the room” during prognostic disclosure:

“What really struck me was how you do feel like you’re living through the process with the family… Living all those intense moments with the family feels extremely different than even what the providers themselves might feel.”

Some participants felt the emotion of experiencing disease reevaluation discussions with the families so intensely that they became uncomfortable and concerned they might be intruding: “In a way, it almost feels like you are listening to a private conversation, like you’re impinging on their privacy.” All participants agreed that reviewing transcripts and recordings represented more than a research task – for many, it felt like an honor to witness families most challenging moments.

### 3.4 Skills for aspiring clinicians: clarity

One working group member, who began qualitative research as an undergraduate student and is currently a medical resident, explained how her prior experiences with qualitative research actively motivate her to be clearer in her communication with patients and families:

“My experience with [reading transcripts] has…informed core beliefs I have regarding communication with patients, especially related to giving bad news… Remembering how [a particular] family felt from not discussing the full extent of the truth encourages me to…talk about all possible outcomes early… It also motivates me to be honest, even when it is hard. So many parents [in interviews]…said they didn’t want someone to ‘beat around the bush.’ I want to tell the truth in a kind way and set the scene for success.”

Another member, who was exposed to qualitative research while practicing as a child life specialist, also underscored how qualitative research training has helped her better understand the value of intentionality when communicating bad news, including exploring and accepting patients’ and families’ reactions to the news conveyed:

“The experience of coding has definitely influenced my clinical practice. One parent…shared that her first thought when our team offered legacy building interventions was: ‘Are you f***ing kidding me?’ I find myself actively thinking about this parent and her reaction every time that I am about to offer these types of interventions – and furthermore thinking about the themes that emerged when coding this data that reiterated the various ways our introduction of these interventions may be improved.”

Several working group members also explained that clinical communication research projects led them to develop heightened awareness of the impact of language on patients/families: “[I developed] awareness that the words that we use can have these long-lasting ramifications and impact. It gives you a heightened cognizance of how important the language and interactions are.”

### 3.5 Skills for aspiring clinicians: compassion

Compassion through understanding and empathy was a pervasive theme across working group members’ reflections on participating in qualitative research: “I think [participation in qualitative research] helps foster empathy and compassion. Medicine can be very draining; there are many systemic barriers to providing care in a patient-centered, thoughtful, and kind way.” Many articulated how qualitative research participation helped them to dig deeper into patients’ and families’ stories, not just limited to clinical encounters in clinical spaces: “Seeing the stories, not just the patients. This work inherently teaches you about the value of the story.” One author explained that this sense of walking with patients and families helped foster patience: “I think [patience] comes from having more perspective into their narrative and giving them the benefit of the doubt because you have not just the hospital, clinical side – you have more insight into the other side of things.” Working group members emphasized how more complex understandings of patients’ and families’ lives generated deeper understanding and compassion, which they recognized as skills integral to provision of high-quality medical care in their future careers.

### 3.6 Unanticipated impact: Self-care and professional affirmation

Working group members identified various unexpected positive outcomes from participation in qualitative research, including how it influenced perceptions of self-care and professional affirmation. Witnessing the strength and wisdom of parents of children with serious illness and/or bereaved parents inspired many learners. One member explained that studying communication through qualitative research methods allowed her to be less harsh on herself in evaluating her own communication: “This experience has provided me with the space to allow myself grace when I know I could have completed an intervention in a different way.” Several also shared that the research experience has affirmed their decisions into go into healthcare professions:

“I remember sitting down and really listening to one of the conversations, and immediately, I was so filled with emotion that tears really filled my eyes, partly because of the emotion of the conversation but also because I had been longing for this viewpoint, as it addressed why I had become really passionate about nursing. It reignited my passion for nursing and healthcare.”

Another echoed this same idea, explaining that the work reinvigorated her medical studies: “Seeing how this life experience affects these parents each and every day really allowed me to see the gravity of the situation and gave me the motivation to continue on this journey towards becoming a doctor.”

An additional unexpected benefit of qualitative research participation was deconstruction of hierarchy in clinical medicine. The process through which team members from various roles and statuses came together to reach consensus in coding belied self-perceived hierarchical identities:

“I was surprised by the richness of diligence and detail involved in the work, including check mechanisms that produced consensus. I found the reconciliation process to be a perfect example of this. The meetings were equal parts presentation of fact and defense of personal standpoint that involved everyone as an equal partnered contributor.”

An author who participated in this research as an undergraduate emphasized the power of connection among team members that overrode the difference of education level or training experience: “We connected on raw emotion that we felt from the conversation – even though we may be at very different stages of life, we still felt the same responses to some scenarios.”

Working group members emphasized that qualitative research participation also helped them develop skills in teaching and mentoring, as well as influenced how they approached the development of communications training programs and curricula in the future. Finally, they explained that it inspired them to continue self-reflection on communication, driving them to develop their practices of life-long learning. Table 3 articulates both benefits in more detail. Alongside these positive benefits, working group members also identified unexpected challenges, acknowledging the emotional weight of accompanying families, bearing witness, and feeling responsible for empathetic and compassionate communication, detailed in Table 4.

**Table 3:** Benefits of qualitative research participation as educators and life-long learners.

Lesson learned	Example quotation
*Educator: teaching and leadership skills development*	
Key examples for teaching and mentoring	• “I talk about this experience as much as possible with peers and mentees to provide a space for further discussion. I have more questions now than I ever have, and it is validating for myself and others to hear that some of our questions/thoughts correlate to one another. I have enjoyed the opportunity to prompt further thought/reflection regarding our current practices and support a space in which we are able to think about why we do things a certain way as well as how we may continue to evolve and improve them.” • “I think it goes back to just giving [bereaved parents] that voice and trying to share their experience of what they go through [with students and trainees], so that mentees and residents can have that insight, a little bit [of] the other side of what they see on rounds.” • “The coding experience has influenced the way in which I communicate and educate families, other clinical staff, and those within the field of Child Life. I thought this data would provide a clear picture of how to work with bereaved families; however, I learned that I have more questions now than I ever have and find myself processing these questions with others often. Thus, I have become more passionate, and it has become more important for me to provide a space to further discuss these concepts with others to understand various perspectives and experiences.”
Ideas for program development	• “Learning more about their grief experience, for example, knowing that the second year can be much more difficult than the first. So making sure that we implement a one-year anniversary card and making sure that we continue that relationship over a longer period of time.”

*Life-long learner: ongoing reflection throughout career*	
Need for ongoing refinement of communication in clinical practice	• “It also reminded me that while I think of myself as a person who is good at communication, I still have much to learn.” • “I find myself reflecting on some of the not-so-great communications scenarios that I have read [in transcripts] when I am communicating, [and] if I start feeling like I am actually using some of that fuzzy or gray language, that’s when I think ‘oh, wait, I read this, and I did not think that was good on paper…’, so I try to redirect what I am saying [in real time]. It just pops into my mind when I hear myself using the language that I didn’t think was great on paper.” • “I reflect on this experience just about every day in my clinical work. The process of having to pause, reflect, and listen to the themes that emerged has encouraged me to take the time to reflect further in regard to my daily interactions with families and other staff.”

**Table 4:** Unexpected challenges of participation in qualitative research: the emotional weight of accompanying families, bearing witness, and feeling responsible for empathetic and compassionate communication.

*Challenge type*	Example quotation	Silver lining
*Emotional intensity of families’ experiences*	• “We were interviewing bereaved parents for our study, and I noticed that it was hard to focus after reading a few at a time. I learned that I had to spread the work out over various days or else I would take on a lot of the parents’ emotions and feelings that were expressed in these interviews.” • “One thing that really surprised me was how engaged I became with the stories of these bereaved parents. I honestly didn't think that I would be able to connect with the transcripts as much as I did. I thought that since I was reading these words through a screen and not living out the patient care experience alongside them, that it wouldn't affect me much. But surprisingly, I found myself shedding tears over the words of these parents. Hearing their stories in their own narrative and hearing about how highly they speak of their children was just so moving in a way I had never experienced. As the parents were describing their own struggles with faith, it made me reflect on my own beliefs and wonder why certain things happen the way they do (and even if a reason for tragedy exists). The transcripts were so powerful that they made me think deeply about our world and if my beliefs need adjusting.”	Learning to anticipate the emotional intensity of clinical encounters for all groups involved, thereby strengthening their emotional intelligence in clinical and research contexts.
*Weight of bearing witness, responsibility of representing families’ perspectives accurately*	• “I wanted to be true to the data, not include my bias… The weight of being a good scientist with the data surprised me.” Another explained a similar responsibility when selecting which quotations to share in formal journal articles: “The qualitative research that gives the family a voice… You feel responsible for that, sharing their narrative. Even when it comes to writing your paper and having to pick one quote out of hundreds…”	Ingraining of research ethics and taking on the responsibility of representation in qualitative research
*Self-critique in moments of less-than-ideal communication (before or after qualitative research experience)*	• “I feel myself getting more frustrated when I don’t know how to say the right thing because I have judged other clinicians. I judge myself much more harshly.” • “I was a nurse for over a decade, and I worked in NICU, so there was a lot of death that we had to deal with because we always did from beginning to end. Looking back now that I am learning more, thinking about things I should have said differently or things I didn’t say that I wish I had said and wishing I had the opportunity to right those wrongs.”	Practice in giving grace to oneself, recognizing that clinicians are humans who make mistakes in communication approaches, and adopting a growth mindset.

## 4 Discussion and conclusion

### 4.1 Discussion

**Fig. 2:**
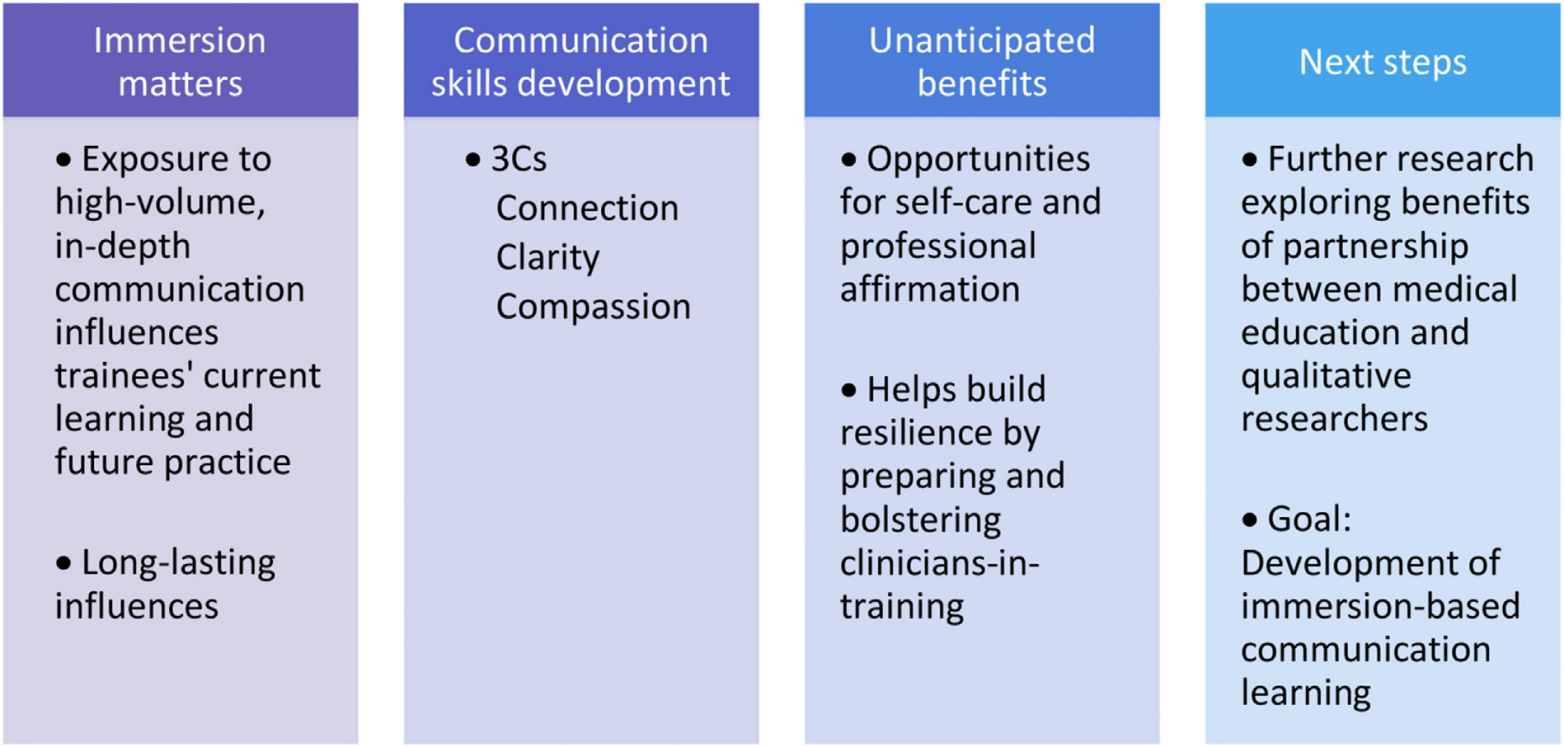
Key takeaway lessons from exploratory investigation of influences of participation in qualitative research on clinicians-in-training.

Clinician-researchers with immersive experience in qualitative research identified the value of research participation on gaining and sustaining important communication skills. Key lessons from the working group are summarized in Fig. 2.

These findings raise the possibility that opportunities for participation in qualitative research alongside communication scientists may be an under-utilized resource for medical educators seeking to support trainees in developing important communication skills. Theories of experiential learning [26,27] and reflexive learning [28,29] underscore the potential educational benefit of qualitative research participation [30], in that they suggest that learning through doing and informally and formally reflecting on experiences may be more effective at conveying key lessons in clinical communication than didactics, small group discussions, or other instruction on the topic. We encourage medical educators and communication researchers to explore strategies for collaboration through engagement of undergraduate, graduate, medical, and post-graduate clinicians- and researchers-in-training. Rethinking the potential of qualitative research to improve clinical education may be bidirectionally beneficial, strengthening communication skills training while also reinforcing the value of qualitative research.

Further, these data preliminarily suggest that regular immersion in qualitative data may support resilience building for learners, and future research is needed to explore this potential. Listening to recordings or reading transcripts from clinical encounters offers trainees a unique opportunity to bear witness and experience diagnostic or prognostic communication, metaphorically standing alongside the patient and family, while still maintaining space to reflect, question, cry, otherwise respond, pause, discuss, and debrief the encounter. DIPEx International and other similar resources amalgamating qualitative research data can be incorporated into learning opportunities that enable more clinical trainees to conduct qualitative research.

The research team functions as a support group within which researchers can process the emotional weight and lessons learned from the encounter. This “practice run” prior to driving difficult conversations offers trainees the chance to develop communications skills and bolster both their approach and confidence prior to patient encounters [31]. Prior qualitative research experiences enable trainees to avoid feeling overwhelmed, hitting the ground running, prepared for the emotional burden and capable of listening, leaving room for silence, building empathy, and prioritizing compassion. Working group members felt prepared not only to practice skillful communication, but also to teach strategies.

Findings from the work should be interpreted in the context of limitations. Working group members all had participated in qualitative research previously and thus likely had a predisposition for engagement with and enthusiasm for communication research and qualitative methodology. It is possible that a different group of learners – perhaps those who tend toward a more positivist sensibility – may not find participation in qualitative research as useful for communication skills development. Additionally, not all QUEST working group members had an opportunity to participate in collective, generative dialogue to build upon ideas in real time. Several members participated by sharing their perspectives in writing, and although this allowed for enrichment of perspectives and experiences, it is not possible to know how additional interaction may have shaped the collective message.

Innovation: We offer two innovative approaches to healthcare professions education. First, we offer an innovative research methodology – an adaptation of autoethnography that involves collaboration among a group of people who share an experience (i.e., qualitative research participation), generate reflective data about that experience, and then work together to analyze those data. The methodology carried out by the collaborative working group to explore an *a priori* question related to our collective experiences is innovative, in that there was no division between “researchers” and “study participants” and thus the process was not traditional “research” but rather collaborative generation of knowledge. Inspired by autoethnographic methods, in which one person generates data from their own experiences, observations, and reflections and then analyzes those data, we have embarked upon a modified autoethnographic endeavor in which we collected data from ourselves as a working group made up of people with shared qualitative communication research participation experience and then analyzed and interpreted those data collectively. Different members of the working group participated in different ways to generate and analyze the data; we generated our own data and then studied our own experiences by analyzing the data. This methodology enables and may even empower health professions educators to study their own educational innovations.

Second, we offer a pedagogical innovation for health professions education, in which participation in qualitative research provides a learning experience for students in the health professions. We found that experience in qualitative research about communication facilitated learning about how to connect with patients and families, communicate clearly, and practice with empathy and compassion. Beyond the communication domain, additional applications of qualitative research experience as a learning opportunity might involve topics such as resilience, mindfulness, meaning-making, and self-reflection as tools to combat burnout or compassion fatigue.

With regards to application of findings, rethinking qualitative research participation as an underutilized educational opportunity is pedagogically innovative and should inspire medical education leaders to collaborate with communication researchers in engagement of undergraduate, graduate, medical, and post-graduate trainees. Collaborations between health professions educators and qualitative researchers could lead beyond communication, expanding to teaching about self-awareness, humility, active listening, quiet observation, and the critical importance of triangulating data to deepen information synthesis and interpretation. Rich opportunities exist to further probe how students immersed in qualitative research gain knowledge and skills. Further research also is needed to explore the benefits of partnerships between medical education and qualitative research teams in development of immersion-based communication learning.

### 4.2 Conclusion

Exposing clinical trainees to communication through participation in qualitative research has the potential to enhance self-perceived communication competency in three key domains: (1) Connection, (2) Clarity, and (3) Compassion, preparing them for future clinical encounters. Further, such exposure may have the potential to strengthen emotional intelligence and promote self-care, professional affirmation, and resilience.

## Funding sources

This research did not receive any specific grant from funding agencies in the public, commercial, or not-for-profit sectors.

## Credit author statement

Amy Porter: Conceptualization, Data curation, Formal analysis, Investigation, Methodology, Project administration, Writing – original draftWriting

Cameka Woods: Data curation, Formal analysis, Investigation, Methodology, Project administration, Writing – review & editing.

Erica Kaye: Conceptualization, Data curation, Formal analysis, Investigation, Methodology, Project administration, Resources, Supervision, Writing – original draft

All other authors: Data curation, Formal analysis, Investigation, Methodology, Writing – review & editing

## Declaration of Competing Interest

None.

## Appendix A Supplementary data

QUEST Working Group Discussion Guide Image 1
