# Supplementary material for: Novel approaches to communication skills development: The untapped potential of qualitative research immersion
Source: PEC Innov. 2022 Aug 26;1:100079. doi: 10.1016/j.pecinn.2022.100079 (PMC10194302; doi:10.1016/j.pecinn.2022.100079)
Supplement: Supplementary file 1 — QUEST Working Group Discussion Guide [file mmc1.pdf]

1. Before you started coding, did you anticipate that the coding experience would have impact on you as a clinician-in-training or in your non-clinical future profession?
    - If so, how? In what ways? Why?

2. When you started coding, did anything surprise you?
    - If so, what?

3. Were there any recordings or transcripts that you struggled with while coding?
    - If so, please describe. How did you handle it?

4. How much, if any, do/did you reflect on your coding experience while coding?
    - If you listened, how did it change you?
    - If you read transcripts, how did it change you?

5. Do you think that doing the other (listening v. reading) would have had the same (or different) impact?

6. Did listening, reading, coding, or analyzing/synthesizing data help you develop any personal or professional skills?

7. For those who are now in clinical training, do you think the coding experience has influenced your clinical practice in any way?
    - Specific stories that illustrate influences?
    - Themes to probe: humanism, resilience, interpersonal relationships/rapport building, communication

8. For those who are doing non-clinical training or work, do you think the coding experience has influenced your training or work in any way?
    - Specific stories that illustrate influences?
    - Themes to probe: humanism, resilience, interpersonal relationships/rapport building, communication

9. How much, if any, do you reflect on your coding experience while doing your current work (either clinical or non-clinical)?

10. Have you shared anything from your coding experience with peers and/or mentees as an opportunity to educate, support, or train others?
